# GNViT- An enhanced image-based groundnut pest classification using Vision Transformer (ViT) model

**DOI:** 10.1371/journal.pone.0301174

**Published:** 2024-03-25

**Authors:** Venkatasaichandrakanth P., Iyapparaja M.

**Affiliations:** School of Computer Science Engineering and Information Systems, Vellore Institute of Technology, Vellore, Tamilnadu, India; Gomal University, PAKISTAN

## Abstract

Crop losses caused by diseases and pests present substantial challenges to global agriculture, with groundnut crops particularly vulnerable to their detrimental effects. This study introduces the Groundnut Vision Transformer (GNViT) model, a novel approach that harnesses a pre-trained Vision Transformer (ViT) on the ImageNet dataset. The primary goal is to detect and classify various pests affecting groundnut crops. Rigorous training and evaluation were conducted using a comprehensive dataset from IP102, encompassing pests such as Thrips, Aphids, Armyworms, and Wireworms. The GNViT model’s effectiveness was assessed using reliability metrics, including the F1-score, recall, and overall accuracy. Data augmentation with GNViT resulted in a significant increase in training accuracy, achieving 99.52%. Comparative analysis highlighted the GNViT model’s superior performance, particularly in accuracy, compared to state-of-the-art methodologies. These findings underscore the potential of deep learning models, such as GNViT, in providing reliable pest classification solutions for groundnut crops. The deployment of advanced technological solutions brings us closer to the overarching goal of reducing crop losses and enhancing global food security for the growing population.

## 1. Introduction

Agriculture is a pivotal industry that sustains human and livestock populations globally, the period from the 1960s to the 1980s witnessed significant advancements in agriculture, commonly referred to as the third agricultural revolution. These advancements included the introduction of improved crop varieties, using synthetic fertilizers and pesticides, and the widespread adoption of irrigation techniques. These innovations played a pivotal role in boosting crop productivity and ensuring food security, particularly in developing nations [1]. However, recent trends indicate a deceleration in agricultural production growth, raising concerns given the escalating challenges like climate change, population boom, and urban migration [2].

Peanuts, commonly referred to as "groundnuts" in certain regions, are the edible seeds of a legume plant. India holds the position as the world’s second largest producer of peanuts. Apart from serving as a dietary staple, this versatile crop significantly contributes to the economic landscape of nations [3]. However, its cultivation is perpetually shadowed by pest threats like Thrips, Aphids, Armyworms, and Wireworms, compromising the yield, product quality, and nutritional value, leading to significant economic repercussions and threatening food security [4]. Further complicating its cultivation is the intricate interplay between the crop’s environment and anatomy. Specifically, conditions such as heightened humidity during the seedling phase foster the swift proliferation of microorganisms, posing additional challenges to maintaining crop health and output [5].

Traditionally, pest and disease identification in agriculture has been anchored in human expertise, a method enriched with invaluable insights but fraught with inefficiencies, significantly when scaled to vast farming landscapes [6]. This reliance often results in time-intensive processes and potential inaccuracies in identifying various pests [7]. Recognizing these limitations, there has been a compelling shift towards harnessing technological advancements, notably computer vision and deep learning techniques, aiming to automate and significantly elevate the precision of pest identification [8].

Emerging technologies such as Deep Neural Networks (DNN), Convolutional Neural Networks (CNN), and Machine Learning (ML) have consistently shown potential to revolutionize pest and disease detection in agriculture [9]. Among these, the Vision Transformer (ViT) has recently gained exceptional prominence, outpacing traditional CNNs in specific applications due to its remarkable performance across diverse computer vision tasks [10]. Especially when ViT models are pre-trained on extensive datasets like ImageNet, they demonstrate impressive transfer learning capabilities tailored to specific domains like agriculture [11]. However, despite these advancements, real-time applicability and optimal accuracy challenges persist, highlighting areas for further exploration and refinement.

Amidst the challenges encountered in groundnut cultivation and the transformative potential of contemporary deep learning techniques, this study introduces the GNViT model to leverage the Vision Transformer (ViT) capabilities for groundnut pest classification. Rooted in the urgency for reliable disease diagnostics and the promise of technological advancements, our research not only aspires to amplify the efficiency of pest detection but also provides a robust solution to the perennial issue of crop loss due to pests, striving for real-time accuracy and reinforcing the imperative of safeguarding our agricultural assets.

The GNViT method is an innovative approach designed to tackle the complexities of early pest prediction in groundnut crops. The process commences with essential data pre-processing steps, followed by data augmentation, which enriches the dataset to accommodate the variety encountered in real-world scenarios. Class labeling is then performed, categorizing the pests as Thrips, Aphids, Armyworm, and Wireworm. The data is systematically divided into training, testing, and validation sets to ensure robust model training and evaluation. The core of this system is the Vision Transformer (ViT) model, adapted from the PyTorch framework. With the computational prowess of GPUs, GNViT initializes with a pre-trained ViT model and tailors its final layer to resonate with the number of pest classes identified. The Cross-Entropy Loss function measures the disparity between predicted and actual outcomes. The Adam optimizer seamlessly handles the optimization with a learning rate of 0.0001. In essence, GNViT encapsulates all necessary stages, from data loading and Augmentation to iterative training loops and final validation, crafting a comprehensive solution to the age-old challenge of pest identification. This method achieved an accuracy rate of 99.52%, a testament to its efficacy, thereby outstripping other prevailing algorithms in the domain. The significant contributions of the research are as follows.

1. This study addresses the limitations observed in CNN-based pest detection and classification models. While CNNs have improved these tasks, they suffer from issues like translation invariance, locality sensitivity, and a lack of global visual understanding. In response, this paper introduces a novel approach that utilizes Vision Transformers (ViT) to enhance and optimize groundnut crop pest classification, overcoming the limitations of CNN-based techniques.
2. The study customizes the ViT model for detecting and classifying groundnut crop pests, drawing inspiration from the work of Dosovitskiy. A et al. [10]. This tailored approach leads to substantial improvements over traditional CNN methods. The model’s accuracy is significantly increased through enhancements to the ViT model’s architecture and the implementation of data transformation augmentation techniques.
3. The proposed GNViT model demonstrates remarkable accuracy in pest detection and classification. This achievement underscores the method’s effectiveness in detecting and classifying groundnut crop pests. Ultimately, this research reduces risks associated with dietary safety and food shortages.

This paper provides a comprehensive overview of the GNViT model, its implementation, and its remarkable results, highlighting its potential to contribute to sustainable and efficient agricultural practices. The paper is structured as follows, In Section 2, we review basic preliminaries and relevant literature related to pest classification on groundnut. Section 3 outlines the materials and methods, Section 4 presents the results obtained from the proposed techniques and discussions. Finally, Section 5 summarizes the findings, offers conclusions, and provides recommendations for future research.

## 2. Basic preliminaries and literature works

### 2.1. Motivation

The motivation for developing the research is rooted in the critical need to improve pest detection in groundnut farming, a key agricultural sector facing substantial yield losses due to pest infestations. Traditional methods of pest detection are labor-intensive, time-consuming, and often inaccurate, leading to delayed or ineffective pest management. This research aims to address these challenges by leveraging the advanced capabilities of Vision Transformer (ViT) models in deep learning. The GNViT model proposes a novel, efficient, and more accurate approach to pest classification, offering a significant step towards enhancing crop management and bolstering food security in regions heavily reliant on groundnut cultivation.

### 2.2. Background

The background of the study is centered around the challenges faced in groundnut agriculture due to pest infestations, a critical issue affecting crop yields and farmers’ livelihoods globally. Groundnuts, a vital source of food and economic stability, are susceptible to various pests, and the inefficacy of traditional pest detection methods exacerbates the problem. The evolution of Artificial Intelligence and deep learning technologies, especially in image processing and recognition, has opened new possibilities for agricultural applications. Vision Transformer (ViT) models, renowned for their success in complex image-based tasks, present an innovative solution. This study aims to explore and validate the application of ViT models in accurately identifying and classifying pests in groundnut crops, addressing a significant gap in precision agriculture, and contributing to more effective and sustainable farming practices.

### 2.3. Literature review

This section explains the theoretical foundation underpinning the introduction and the application that will be explored. It also describes the relationship between these concepts and their practical application. One of the most crucial domains in machine vision is the identification and analysis of pests and diseases affecting crops. In the context of pest detection and classification, this approach employs computer vision equipment to capture images of grain crops [12]. The agricultural sector has been at the forefront of adopting computer vision-based technology for crop diagnosis and pest identification, partly replacing conventional farming practices for pest classification. Traditional methods for pest detection in groundnut crops heavily relied on conventional image processing algorithms, often accompanied by manually crafted features aimed at capturing specific disease-related characteristics. However, these methods had inherent limitations. Unpredictable natural environments with changing light conditions, varying shadows, and inconsistent background textures posed significant challenges. The dynamic nature of these environmental factors made traditional methods resource-intensive and less effective in ensuring accurate pest detection despite their focus on plant pathologies for classification [13]. Automation in agriculture has advanced significantly in recent years because of robotic devices and artificial intelligence. Counting fruits, weed/crop discrimination, plant disease identification, and land cover categorization are just a few of the agricultural activities that have been transformed by machine learning and deep learning approaches, especially convolutional neural networks. Research has indicated that deep learning models outperform conventional machine learning algorithms, exhibiting significant gains in accuracy rates for tasks like weed/crop discrimination and plant disease detection. These developments demonstrate the possibility of future innovation and automation in the agricultural sector [14].

The rise and widespread adoption of deep learning-based computer vision systems are evident across various industries. Deep learning has revolutionized multiple domains, from enhancing cinematic experiences in the film industry to underpinning foundational technologies in autonomous driving. Given the economic importance of groundnut crops, advancements in early pest detection have become particularly relevant. Timely detection impacts crop health and significantly influences market prices due to the direct link between yield and quality. In this context, Artificial Intelligence, specifically deep learning techniques, has introduced transformative innovations in Precision Agriculture [15]. The authors of [16] implemented a deep convolutional neural network (DCNN) to address the challenges of detecting and classifying diseases in groundnut plants. They collected groundnut leaf disease images from the Plant Village dataset and trained the DCNN using the stochastic gradient descent momentum method. Through rigorous testing, mainly focusing on the 6th combined layer of the DCNN, they achieved a remarkable accuracy rate of 95.28%. Ultimately, their proposed DCNN model demonstrated an outstanding overall accuracy of 99.88% in classifying groundnut diseases.

Recent advancements have highlighted the utilization of hyperspectral imaging in insect detection [17]. Similarly, a comprehensive study [18] synthesized findings from various computer vision-based systems explicitly designed for monitoring pests in agricultural settings. Another scholarly endeavor [19] delved deeper into this realm, evaluating multiple machine learning-driven strategies for pest detection and intriguingly proposed a deep learning technique that leverages spectral signatures to enhance pest classification. Transfer learning relies on training a model with one dataset and deploying its acquired knowledge on an entirely different dataset. This significantly shortens the learning curve and bridges the gap between training and testing datasets [20]. The authors of [21] propose a method for automated pest detection in agriculture due to labor-intensive manual methods. Evaluating nine deep learning algorithms, ResNet-50 emerged with a remarkable 99.40% accuracy in pest classification, marking a significant stride in precision agriculture. In [22], the authors develop a deep learning model based on CNNs for diagnosing and classifying plant diseases. They tested their model on a dataset of images for 14 different plant diseases, achieving an accuracy of 98.38%. This work emphasizes the effectiveness of deep learning for automated plant disease detection.

The authors of [23] discuss Image Transformers (ViT), a novel architecture in computer vision with the potential for crop disease classification. While these papers do not explicitly address crop diseases, they showcase ViT’s ability to capture long-range dependencies in images, potentially improving disease detection compared to traditional CNNs. However, research directly applying ViT to this task with relevant datasets is necessary to evaluate its efficacy in crop disease classification. In the paper [24], the authors proposed a LeViT, a hybrid architecture that combines CNNs and Transformers to achieve faster inference speed while maintaining competitive accuracy. The paper [25] introduces the Tokens-to-Token Vision Transformer (T2T-ViT) model, which is trained from scratch on the ImageNet dataset. The main idea behind T2T-ViT is to convert image patches directly into tokens, similar to how words are tokenized in natural language processing tasks. This approach simplifies the training process of Vision Transformers by eliminating the need for patch embeddings, leading to better scalability and efficiency.

In a series of diverse studies employing computer vision and machine learning, the research presents advance-ments and specific outcomes across various domains. The authors of [26] introduce an optimized cascaded graph convolutional neural network for carrot grading by extracting different features, yielding enhanced automation in agricultural processes. The authors of [27] focus on early disease detection in tomato plants utilizes machine learning techniques, including Support Vector Machine (SVM), Convolutional Neural Network (CNN), and K-Nearest Neighbor (K-NN), are employed for classification, achieving notable accuracy rates of 88% (SVM), 97% (K-NN), and 99.6% (CNN). These studies collectively underscore the impactful outcomes and versatility of computer vision models in addressing specific challenges across agriculture, plant health, image segmentation, and combustion monitoring.

Groundnuts, a vital crop in the Indian agricultural landscape, unfortunately, face numerous diseases. These maladies compromise the crop’s health, negatively impact yield and quality, and have economic ramifications [28]. While multiple research efforts have harnessed Convolutional Neural Networks (CNNs) to address pest identification challenges in crops, many have limitations in scope and generalizability, often tailored for specific crops and pest varieties [29]. The authors [30] address the food security challenges through automated disease detection in cultivated land. Traditional manual scouting methods are inefficient and error-prone, prompting the adoption of advanced image processing techniques and deep learning algorithms for accurate disease identification. The study employs Faster R-CNN with ResNet50 to detect and classify tomato diseases, showcasing promising results in complex plant environments. Several research endeavors have used extensive image datasets for pest identification, yet real-time detection challenges and variations in experimental conditions remain persistent barriers [31]. Vision Transformers (ViTs) have made strides in image classification but encounter efficiency bottlenecks, especially when trained on mid-sized datasets like ImageNet. Innovative approaches to enhance ViTs, such as the Token-to-Token vision transformer, still exhibit inherent constraints [32]. Another concern is ViTs’ susceptibility to adversarial attacks, potentially compromising their robustness [33]. This study [34] tackles the challenge of classifying images with multiple spectral bands, which is crucial in remote sensing and medical imaging. The authors propose a unique approach: combining an "adaptive spectral-spatial kernel" with an improved ViT. This kernel extracts critical features from spatial and spectral information, feeding them to the ViT for precise classification. Their method outperforms existing techniques on various datasets, demonstrating the potential of ViTs for specialized image analysis. This work [35] delves into standard RGB image classification, aiming for higher accuracy across diverse datasets. The authors introduce "CrossViT," a multi-scale ViT architecture that leverages "cross-attention" mechanisms. CrossViT extracts features at different scales within an image and intelligently links them using cross-attention, leading to a more comprehensive understanding of the image content. This innovative approach achieves state-of-the-art results on several benchmarks, suggesting the potential of ViTs for general image classification tasks. Given this landscape, a compelling need is to develop a more adept Vision Transformer model seamlessly integrated with CNN paradigms to revolutionize pest identification in the groundnut agricultural ecosystem. A review of the literature on the use of Vision Transformer models for groundnut pest classification reveals that enhanced deep transfer learning models can significantly improve the accuracy and efficiency of the classification process.

Furthermore, the application of Vision Transformer models can help overcome the challenges of detecting and classifying pests in leaves within their dynamic environments. These related works offer insights, benchmarks, and contextual background for the proposed GNViT approach and pest classification performance. This background helps position the research’s novelty and significance within the broader domain of agricultural applications and deep learning techniques.

## 3. Materials and methods

The experimental dataset used in the investigation is described at the outset of this section. The pest detection model, known as GNViT, is then introduced. The experimental setting and assessment measures must be studied to assess the model’s effectiveness.

### 3.1. Groundnut pest insect dataset description

Groundnut (peanut) is crucial for many countries from both an economic and nutritional perspective. However, its cultivation is threatened by various pests that can significantly reduce yields and affect the quality of the produce. High-quality images of diverse groundnut pests were collected from the IP102 dataset [36]. These images include Thrips, Aphids, Armyworms, and Wireworms, which are some of the major pests commonly affecting groundnut crops. This research work gathered data from four different insect datasets. The Aphids dataset, with 2456 pest images, is the most significant among them and the most important dataset affecting groundnut crops. The next most severe threat to groundnut crops is Armyworm, which contains 642 pest images. Wireworms, another category of pests, were examined with 532 pest images used for model training and testing. The final groundnut pest considered is Thrips, represented by 527 pest images. The dataset comprises 4157 pest images of groundnut crops categorized into four classes. The data split ratio between training, validation, and testing is 80:20.

Farmers and agriculturalists must remain vigilant against various pests that threaten groundnut crops, including Aphids, Thrips, Wireworms, and Armyworms. Integrated Pest Management (IPM) techniques effectively mitigate these threats by combining cultural, biological, and chemical practices [37]. Alongside traditional strategies, the advent of deep learning models promises timely detection and precise classification of these pests. Such technological advancements aid in executing prompt and specific pest control interventions, ensuring minimal crop damage and optimal yield outcomes.

### 3.2. GNViT–Groundnut pest classification using vision transformer (ViT) model

**Fig 1:**
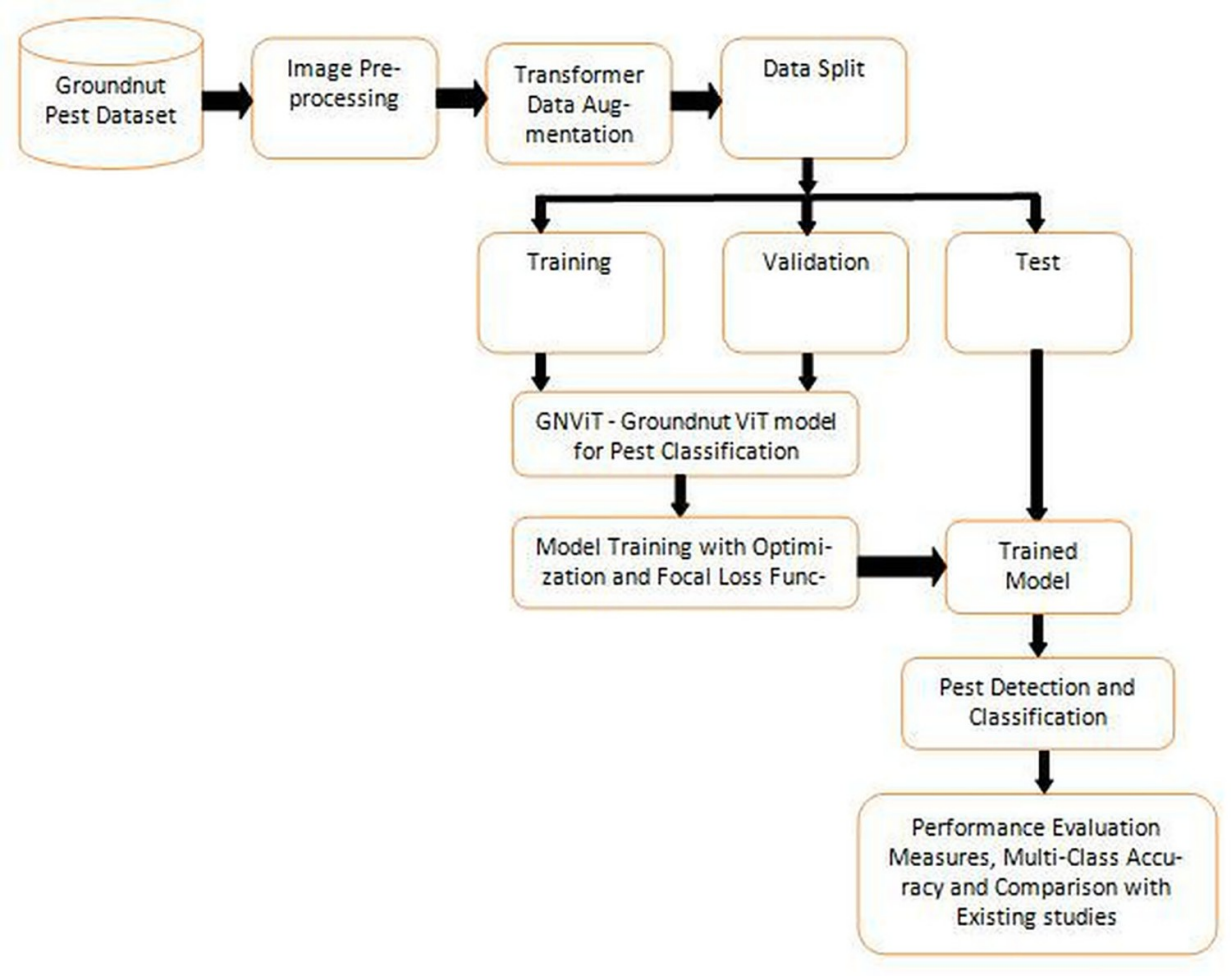
Flowchart for the proposed GNViT–Groundnut crop pest classification using Vision Transformer (ViT) model.

Vision transformer methods have been extensively researched for identifying and categorizing groundnut pests. However, these methods come with several complications, including the potential for misdiagnosis due to variations in leaves, varieties, and environmental factors. Early identification and treatment of pests are crucial, but the lack of agricultural expertise in rural areas can be time-consuming and hindered. With autonomous feature selection and reduced reliance on labor-intensive Image pre-processing, convolutional neural networks have proven effective in image-based recognition tasks. However, one of the challenges is the availability of large and diverse datasets to train these models effectively, which remains a difficult task. Table 1 presents the generalized pseudo code for the GNViT Model, utilizing advances in vision transformer learning models to predict accuracy. Fig 1 represents the flowchart for the proposed GNViT–Groundnut Pest Classification Using Vision Transformer (ViT) Model. GNViT, a novel deep-learning model designed for pest detection and classification in groundnut crops. In this study, the dataset comprises a diverse collection of groundnut crop images, encompassing various growth stages and pest infestation scenarios. The dataset undergoes meticulous preprocessing, including standardization of dimensions and adjustments for brightness and contrast. Error Level Analysis (ELA) is employed for authenticity assessment, ensuring the integrity of the dataset. Data augmentation techniques diversify the training dataset with transformations like rotation and scaling. The augmented dataset is strategically split into training, validation, and testing sets. The Vision Transformer (ViT) model, pre-trained on ViTBase-16 ImageNet-21k, is employed for groundnut pest classification. Model training involves optimization and focal loss to address class imbalance and performance evaluation metrics are computed on the validation set. After training for multiple epochs, the model achieves impressive accuracy on the training, validation, and test sets, outperforming other models. The model’s real-world applicability is tested on the designated testing set and predicted images are scrutinized to assess its proficiency in accurately classifying groundnut pests. This comprehensive methodology ensures robust model training, evaluation, and validation for effective groundnut pest classification. These positions GNViT as a promising tool for accurate agricultural groundnut pest prediction and classification compared to state-of-the-art models. Table 1 presents the generalized pseudo code for the GNViT Model, utilizing advances in vision transformer learning models to predict accuracy.

**Table 1:** The generalized pseudo code for the GNViT model.

Input: Groundnut Pest Dataset Output: Pest Detection and Classification Procedure GNViT: 1. Divide the input image into fixed-size patches. 2. Flatten and linearly embed each patch into a D-dimensional vector. 3. Add a learnable ’class’ token to the start of the patch sequence. 4. Add positional embeddings to each patch embedding. For each Transformer Block in GNViT: 1. Multi-head Self Attention: Compute the query, key, and value for each head. Compute attention scores using the dot product of query and key. Apply softmax to the attention scores. d. Compute the weighted sum of values based on attention scores. e. Concatenate outputs of all heads. 2. Apply Group Normalization (instead of Layer Normalization in the original ViT). 3. Feed-Forward Neural Network: a. Apply a linear transformation followed by a non-linear activation (e.g., GELU). b. Apply another linear transformation. 4. Apply Group Normalization again (assuming it is used both post-attention and post-FFN). 5. Pool the output sequence to get the representation of the ’class’ token. 6. Apply a final linear classifier to get predictions. End Procedure

#### 3.2.1. Image pre-processing and error level analysis (ELA)

Image pre-processing techniques were utilized to enhance groundnut crop pest image classification accuracy, necessitating a large dataset to prevent overfitting during CNN model training. Employing image-preprocessing methods isolates diseased leaves, improving recognition accuracy. Image processing is crucial for pest detection/classification using deep learning, with various techniques enhancing model accuracy [38]. Image compression techniques can reduce the size of pest images without significantly losing important information. Compression methods like Huffman and Run-Length Encoding is a lossless compression [39] method that preserves image details, aiding in accurate pest identification. Hybrid compression [40] combines lossless and lossy methods, balancing compression efficiency and image fidelity.

At first, the training and test photos underwent pre-processing to improve contrast and resize them to a 224x224 pixel resolution. For resizing the images, nearest neighbor interpolation (Eq 1) and for rescaling, the traditional methods (Eq 2 and Eq 3) are used along with image crop.


$$NewPixelValue=(x^{\prime },y^{\prime })=PixelValue(round(x),round(y))$$ (1)

NewWidth=ScaleFactor*OriginalWidth (2)

NewHeight=ScaleFactor*OriginalHeight (3)

Pest images in RGB are converted to grayscale for edge detection. External patterns in pest images are equally perceived from the edge. The region of interest is determined by coordinates (p+r, q+s) and isolated from the primary RGB image, aiding in pest detection and removal.

Error Level Analysis (ELA) is a technique used to analyze digital images to detect potential areas of manipulation or tampering [41]. The primary function of ELA is to highlight regions of an image that may have undergone different compression levels compared to other parts of the Image as defined in (Eq 4). This disparity in compression can indicate potential areas of interest for further investigation, as these regions might have been altered or edited.


ELA=||I_original−I_compressed|| (4)

ELA is the error level of a pixel, representing the difference in intensity values between the original and compressed versions of the Image.

• I_original is the intensity value of the pixel in the original Image.

• I_compressed is the pixel’s intensity value in the compressed image version.

The ELA procedure is performed as follows.

1. Take the original Image and make a copy.
2. Compress the copied Image using a lossy compression algorithm, such as JPEG, at a specified compression quality.
3. Subtract the pixel intensity values of each corresponding pixel in the original and compressed images to compute the error level of each pixel.
4. Apply a color map to visualize the error levels as a color-coded representation, highlighting regions with different error levels and indicating potential areas of manipulation.

#### 3.2.2. Data augmentation and data split

The data augmentation process can be incorporated using PyTorch’s torch vision module [42]. During training, images undergo several transformations to introduce variability and improve generalization. These transformations include random horizontal flipping, a random rotation of up to 10 degrees, random resizing and cropping to a dimension of 224x224 pixels, and color adjustments for brightness, contrast, saturation, and hue. After these augmentations, the images are converted into tensor format and normalized based on mean and standard deviations from the ImageNet dataset. For the validation dataset, the process is more streamlined. Images are resized to 256x256 pixels, then a centered crop to 224x224, converted to tensors and normalized like the training images.

*3*.*2*.*2*.*1*. *Types of transformations*. Data augmentation techniques employed for this research encompassed spatial and pixel-level modifications executed using the PyTorch Torch Vision toolkit. For a comprehensive understanding of these techniques and their application, refer to the Torch Vision official guide [42].

- Spatial Adjustments

Spatial adjustments play a crucial role in diversifying and augmenting the dataset for improved model training. Adaptive resizing and cropping involve randomly selecting and trimming image segments to a standardized size of 224 × 224 pixels. Randomized horizontal and vertical flipping introduce mirroring along their respective axes, enhancing orientation variations. Additionally, arbitrary rotation allows images to undergo random rotations of up to 30 degrees, simulating real-world scenarios. These spatial adjustments contribute to a more comprehensive and varied dataset, fostering the model’s ability to generalize and perform effectively across diverse image conditions.

- Pixel-centric Modifications

Pixel-centric modifications are employed to diversify the dataset and enhance the model’s adaptability to various visual conditions. Chromatic alteration introduces random color variations with specific configurations for brightness, contrast, saturation, and hue. The intentional blurring technique, using Gaussian blur with a kernel size of 0.3, deliberately adds fuzziness to the images. These modifications contribute to a more comprehensive dataset, simulating diverse visual scenarios and improving the model’s ability to generalize and perform effectively across a range of image qualities and conditions.

The Groundnut pest dataset was split into training (80%), testing (20%) sets. Data augmentation techniques were applied to the training set, including rescaling, rotating, shearing, zooming, flipping, adjusting brightness, and modifying channels, to reduce overfitting and increase dataset variation. Adam optimization with forward stature improved ViT model predictions, ensuring output accuracy. The validation and testing sets included images of Aphids, Thrips, Armyworms, and Wireworms, accounting for twenty percent of the training set. The GNViT model successfully categorized images and predicted class labels on the training dataset.

#### 3.2.3. GNViT—Vision transformer model for groundnut crop pest classification

**Fig 2:**
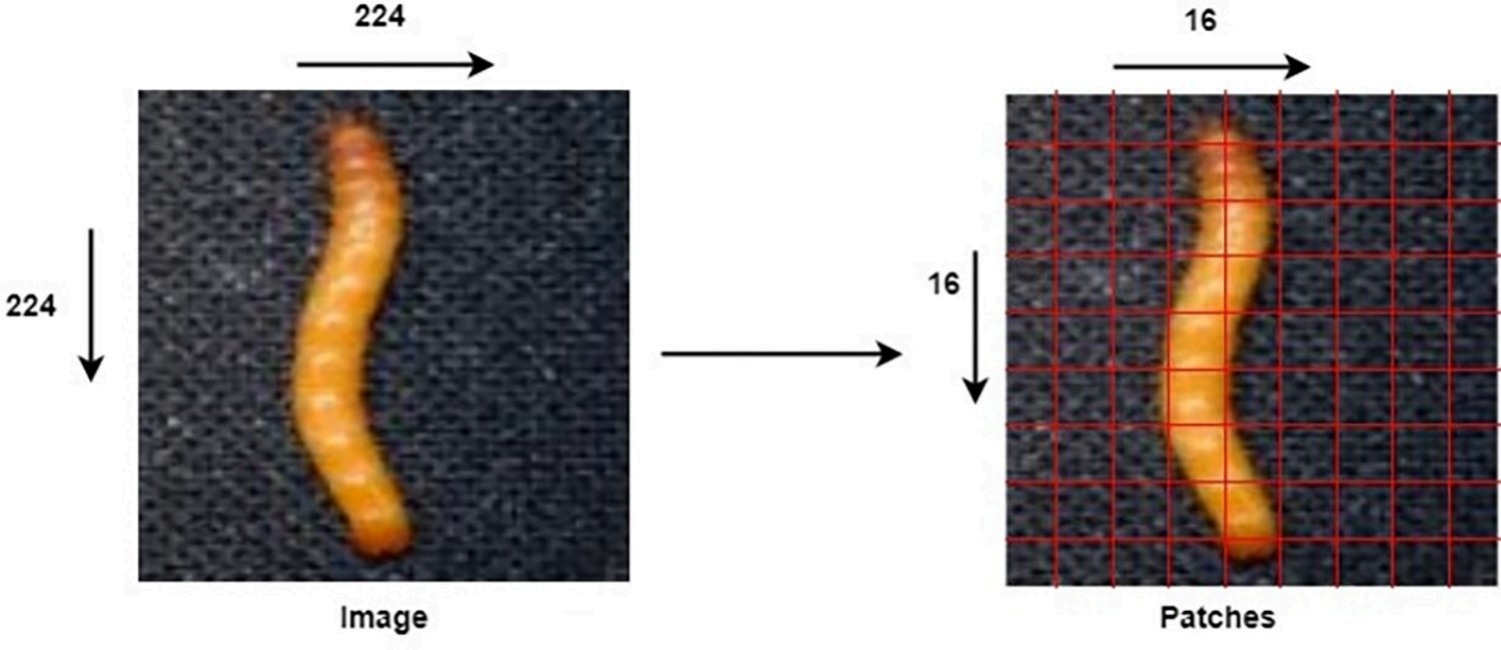
Showing a single image becomes 256 image patches—Republished from [45] under a CC BY license, with permission from [MDPI], original copyright [2022].

Dosovitskiy and colleagues investigated the potential of transformers in image classification, leading to the concept of the vision transformer [10]. Similarities exist between how transformers handle natural language data and the operations of vision transformers [43]. Just as sentences are segmented into individual words, the vision transformer divides images into smaller patches [43]. Patch alignment is crucial to prevent distortion of the entire image [43]. The model developed by Dosovitskiy et al. is tailored for input images of dimensions 224 × 224, which conveniently divides into 256 distinct patches, each measuring 14 × 14 pixels [44]. For the visual illustration presented in Fig 2, which taken reference from the source [45].

**Fig 3:**
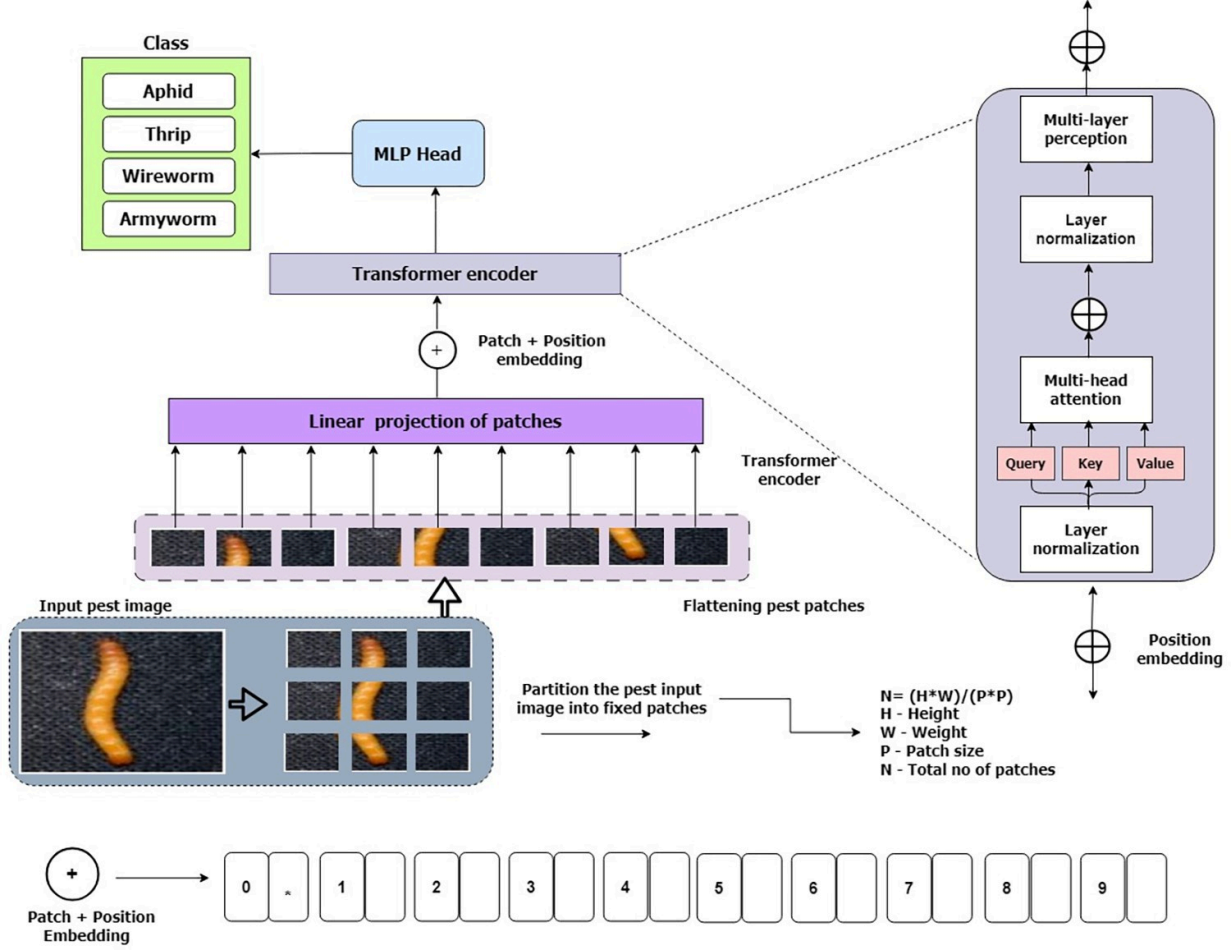
An overview of the proposed GNViT framework for identifying groundnut crop pests—Republished from [45, 46] under a CC BY license, with permission from [MDPI], original copyright [2022, 2023]. The (*) notation appended to the patch + position embedding signifies the presence of a class token, which serves as a pivotal element encapsulating comprehensive image information within the sequence of patch embeddings.

After dividing the image into 256 patches, a linear projection layer preserves their dimensions to ensure equivalent areas receive the same embeddings [43]. The vision transformer incorporates an additional class embedding, denoted as 0, marking the image’s beginning and subsequent patch sequence [10]. This adaptive class embedding aids in creating unique embeddings tailored to each classification tag [43]. Positional markers are added to pre-serve patch sequence arrangement, represented as vectors with dimensions matching patch embeddings, learned during pre-training and fine-tuning [43]. Patch embeddings, along with classification label and positional embeddings are summed and fed into a transformer encoder for image recognition [44]. For visual representation presented in Fig 3, which taken reference from the source [46].

*3*.*2*.*3*.*1*. *ViT model pre-trained on ImageNet-21k*. To do this, we will use the vision transformer proposed by Dosovitskiy et al. and described in full in their paper [10]. They plan to use the Hugging Face ecosystem, particularly the Python ’transforms’ package, to gain access to the structural specifications for their ViT model [44]. The ImageNet-21k dataset, an extensive collection with 14 million images spanning 21k categories, was used to pre-train this vision transform [44]. Notably, this model’s pre-training was conducted on images of resolution 224 × 224 pixels. Consequently, any subsequent training on this model requires the data to adhere to this pixel dimension [44].

This GNViT model applies the transformer mechanism to vision tasks by first converting images into a sequence of patches and then processing these patches with the transformer blocks. The idea is to allow the model to attend to different parts of the Image, like how transformer models attend to different parts of a text sequence in NLP tasks.

1. Patch Embed: The typical transformer processes inputs as a 1D sequence of token embeddings. To accommodate 2D images, we transform the image x, which has dimensions R^ {H×W×C}, into a series of linearized 2D patches. This module breaks down the input image into fixed-size patches and then linearly embeds them into a specified feature dimension [10].

Flattened Patches’ Linear Transformation: Before introducing the patches to the Transformer structure, the authors deemed a linear projection of these patches beneficial. A 2D convolution layer that both breaks the Image into patches and embeds them. Here, it uses a 16x16 kernel with a stride of 16x16 [10].

`norm`: An identity layer means no normalization is applied at this stage [10].

2. Dropout and Identity Layers:

`pos_drop`: A dropout layer, but dropout probability is set to 0, meaning no dropout is applied.

`patch_drop`and `norm_pre`: Identity layers, meaning they do not modify the data.

3. The Transformer Encoder Block: The Transformer encoder is structured with alternating layers of Multi-headed self-attention mechanisms and MLP units. Layer Normalization (Layernorm) is incorporated before each block, and a residual connection follows every such block [44].

Blocks: This is a sequential set of identical transformer blocks. The GNViT has 12 identical blocks, each with the following.

`norm1`: Layer normalization before the attention mechanism in (Eq 5).

$$LN(x)=\frac{x-\mu (x)}{\sigma (x)}$$ (5)

Where μ(x) and σ(x) are the mean and standard deviation of x respectively.

`attn`: The self-attention mechanism, a key transformer component. Compute self-attention scores and apply softmax. For each head, i: Compute query (Eq 6), key(Eq 7), and value matrices(Eq 8):

$$Q_{i}={LN}_{\left(zt-1\right)}\;W_{i}^{Q}$$ (6)

$$K_{i}={LN}_{\left(zt-1\right)}\;W_{i}^{k}$$ (7)

$$V_{i}={LN}_{\left(zt-1\right)}W_{i}^{v}$$ (8)

- `qkv`: A linear layer to derive query, key, and value matrices.
- `attn_drop`: Dropout applied after the attention scores are calculated.
- `proj`: A linear layer that projects the output of the attention mechanism.
- `proj_drop`: Dropout applied after the projection.
- `norm2`: Another layer of normalization before the MLP.
- `mlp`: A feed-forward neural network.
- `fc1`: First fully connected layer.
- `act`: Activation function, in this case, GELU.

Given zt, the MLP typically consists of two fully connected layers with a GELU (Eq 9 and Eq 10) (or ReLU) activation function in between:

$$M1={GELU}_{\left(ztw1+b1\right)}$$ (9)

$$M2={M1}_{\left(ztw2+b2\right)}$$ (10)

- Residual Connection and Layer Normalization is defined in (Eq 11).

$$y={LN}_{\left(zt+M2\right)}$$ (11)

drop1`: Dropout applied after the activation.

`fc2`: Second fully connected layer.

`drop2`: Dropout applied after the second fully connected layer.

The proposed GNViT model will have 12 blocks for encoding and will classify the output into 4 categories.

#### 3.2.4. Optimizers adopted

In developing the model, three optimizers were used to compare the results [47]. The following optimizers were used

- Adam
- RMSProp
- Stochastic Gradient Descent (SGD)

*3*.*2*.*4*.*1*. *Adam*. Adam is an optimization algorithm commonly used as an alternative to standard stochastic gradient descent (SGD) in neural networks for weight updates during training [47]. Adam is a combination of two enhancements to SGD. Specifically, it incorporates the benefits of the "Adaptive Gradient Algorithm" (AdaGrad) to manage the learning rate on a per-parameter basis, which improves efficiency when dealing with gradients that are sparsely distributed in applications like computer vision and NLP, among others.

*3*.*2*.*4*.*2*. *RMSProp*. Another optimization approach, root-mean-square propagation (RMSProp), has a learning rate adjusted for parameters [46]. The ’running average’ is calculated as follows (Eq 12):

$$(w,t)≔(w,t-1)+(1-\gamma ){\left(\nabla Q_{i}(w)\right)}^{2}$$ (12)

The learning parameters are updated as follows (Eq 13): η

$$W≔W-\frac{n}{\sqrt{v(w,t)}}\nabla Q_{i}(w)$$ (13)

Here, "w" is estimated, which minimizes Q. Because it is the iterative method, it performs the following iterations to minimize the objective function.

*3*.*2*.*4*.*3*. *Stochastic gradient descent (SGD)*. It is an ’iterative method’ that optimizes the loss function with differentiable properties [47]. The goal of machine learning is to optimize the loss function or objective function. Mathematically represented in (Eq 14):

$$Q(w)=\frac{1}{n}\;\sum_{i-n}^{n}Q_{i\;}(W)$$ (14)

Here, "w" is estimated, which minimizes Q. Because it is the iterative method, it performs the following iterations to minimize the objective function is defined in (Eq 15).

$$w≔w-\eta \nabla Q_{i}(w)$$ (15)

Where η is the learning rate.

*3*.*2*.*4*.*4*. *Loss function*. The loss function in deep learning is used to quantify the error between the model’s predictions and the actual values to inform its iterative refinement. During training, you should strive to reduce this loss value to its minimum. Based on the nature of the issue, several loss functions may be used. Standard loss functions include the mean absolute error, cross-entropy, and the mean square error. The selection between these loss functions is based on the task at hand and the intended behavior of the model.

The data was highly imbalanced, so using categorical cross entropy could have been more efficient; the other solution was to use weighted categorical cross entropy, where the classes with fewer examples are given more weightage than those with more examples. This technique is also efficient, but the "focal loss" [48] introduced to address the class imbalance problem was more efficient. The focal loss for the binary case can be defined (Eq 16) as,

FL(pt)=−(1−pt)γlog(pt) (16)

Where γ ≥ 0 and is termed as "focusing parameter" and

$$pt=\left\{ \begin{array}{c} p,y=1\\ 1-p,Otherwise \end{array}\right.$$

When any input image is not classified correctly, the term pt moves to 0, and the adjusting factor will become 1, so the loss function is unaffected. If the input image is classified correctly, pt will be 1, and the adjusting factor will be 0. It will make the loss value near 0, which will "down-weight" the input image. The focusing parameter γ regulates the rate at which the input images that are easily classified are down-weighted.

#### 3.2.5. Evaluation metrics used to evaluate the model performance

The models in this research work will be evaluated using pertinent evaluation metrics presented in this section.

*3*.*2*.*5*.*1*. *Accuracy*. Accuracy is also an evaluation metric that is used for the evaluation of classification models. The accuracy value represents the fraction of predictions the model predicts correctly [49]. The formula for accuracy is defined in (Eq 17).


$$Accuracy=\frac{\left(Total\;Number\;of\;Correct\;Predictions\right)}{\left(Total\;Predictions\right)}$$ (17)

*3*.*2*.*5*.*2*. *Precision*. Precision indicates the fraction of correct optimistic predictions [49]. The formula of precision is defined in (Eq 18).


$$Precision=\frac{\left(True\;Positive\right)}{\left(True\;Positive+False\;Positive\right)}$$ (18)

*3*.*2*.*5*.*3*. *Recall*. Recall indicates a fraction of actual positives predicted correctly [49]. The formula of recall is defined in (Eq 19).


$$Recall=\frac{\left(True\;Positive\right)}{\left(True\;Positive+False\;Negative\right)}$$ (19)

*3*.*2*.*5*.*4*. *F1-Score*. It shows the balance between Recall and Precision [49]. The formula of the F1-Score is defined in (Eq 20).


$$F1-Score=2*\;\frac{\left(Precision*Recall\right)}{\left(Precision+Recall\right)}$$ (20)

## 4. Results and discussion

Python libraries implement the proposed research, Pytorch [42], and Vision Transformers for the ViT models experiments presented [10]. It employed the Adam optimizer for training, which possessed a learning rate and a built-in loss function. The experimental analysis is done on a GPU-enabled P-100 server with an i5 processor and 8GB RAM. This research partitioned data into 80% for training and validation and the remaining 10% allocated for testing. The model underwent training for 10 epochs, utilizing batches of 64 samples. The model’s hyper parameters included a set learning rate of 0.0001 and a preliminary warm-up ratio of 0.1. In addition to the methodologies described, our research incorporates error level analysis to assess the robustness and reliability of the proposed model. Error level analysis involves evaluating the model’s performance by examining the errors it produces under various conditions, enabling us to identify and address potential weaknesses in the model’s learning process. This analysis is crucial for refining the model, as it provides insights into how the model behaves with unseen data and under different scenarios. By systematically analyzing the errors made during the testing phase, which comprises 10% of the partitioned data, we gain valuable feedback on the model’s generalization capabilities. This feedback is instrumental in adjusting the model’s hyper parameters and training strategies, such as the learning rate and warm-up ratio, to enhance its performance. The error level analysis, therefore, plays a pivotal role in our experimental framework, ensuring that the model not only achieves high accuracy but also maintains consistency and reliability across diverse datasets. The choice of hyper parameters often involves experimentation and tuning based on the performance of the model on a validation set. The hyper parameter values result in a model that generalizes well to unseen data. The hyper parameter values for the GNViT model were determined through a process of careful tuning. The model was optimized using the Adam optimizer to enhance performance. This careful selection of hyper parameters, in conjunction with the use of data augmentation, contributed to a significant increase in training accuracy, which rose from 55.37% in the initial epoch to 99.52% by the tenth epoch.

The outcomes of the proposed GNViT model revolved around several key aspects.

1. Distinguishing Pest images into multi-pests and predicting into groups for Aphids, Thrips, Armyworms, and Wireworms.
2. Examining the GNViT model’s performance on the New Groundnut pest dataset using Transformer image augmentation methods in the training set and testing.
3. Identifying and evaluating the significant pest categories using test predictions.
4. Assessing the outcomes in comparison to other cutting-edge, contemporary networks.
5. Analyzing the outcomes of experiments using Vision transformers learning to identify pest disease in contrast to earlier research.

### 4.1. GNViT learning model parameters to assess the performance on groundnut crop pest dataset

GNViT trained on pest dataset accuracy and loss. Table 2 illustrates GNViT configurations, epochs, and other parameters. The dataset shows the GNViT model, which changes internal parameters to improve performance during training. The model learns to extract important traits and characterize disease classes over several epochs. Each epoch’s accuracy and loss statistics show model performance. Accuracy is the percentage of correctly predicted instances concerning actual.

**Table 2:** GNViT learning-model-training parameters.

S.No	Parameter used	Value
1	Training Epochs	10
2	Optimizer	Adam
3	Learning Rate	0.0001
4	Batch Size	64
5	Drop out	0.0
6	Image size	224x224
8	Early stopping	Yes
9	Reduce LR	Yes
10	Data Shuffle	True
11	No of Classes	4
12	Callback	True on model checkpoint
13	Loss function	Cross entropy Loss and Focal Loss
14	Activation Function	GELU
15	Weight Decay	1e-4

### 4.2. Performance analysis of the GNViT model on pest dataset with Augmentation

**Fig 4:**
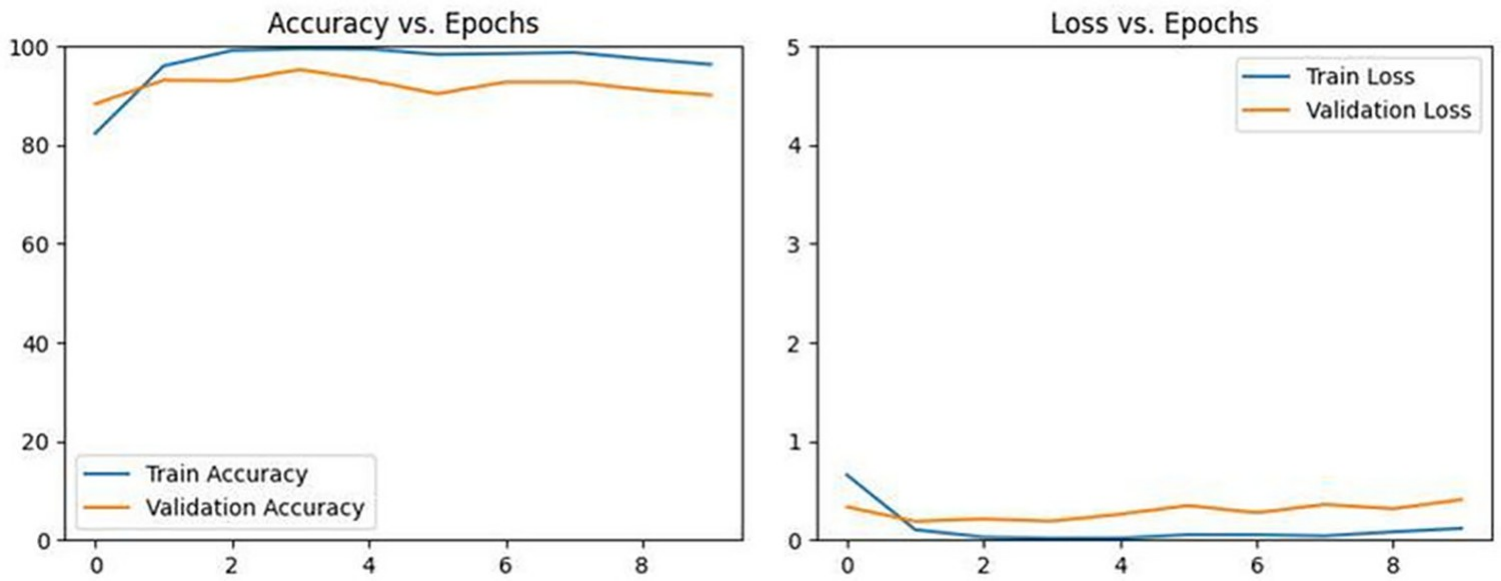
GNViT model performance on groundnut pest classification accuracy for 10 epochs with Augmentation.

When data augmentation is introduced to the GNViT model, a marked performance improvement is observed over ten epochs. Fig 4 describes GNViT model performance on Groundnut pest classification accuracy for 10 epochs with Augmentation. The metrics derived from training and validation datasets display a notable rise in accuracy and a decline in loss. Initially, the model begins with a training accuracy of 83.13% in the first epoch, but at the end of the 10th epoch, this leaps to an impressive 99.52%. Validation accuracy also boosts, reaching as high as 89.18% in the 9th epoch. Despite the surge in performance, the increase in validation loss in some epochs, like the 10th, suggests the model might be overfitting, necessitating potential regularization or early stopping strategies.

#### GNViT model training for groundnut pest classification

Refer to Table 3 to view the performance metrics of the GNViT model on the Groundnut dataset for pest image categorization. The table outlines the Training Loss, Training Accuracy, Validation Loss, and Validation Accuracy across 10 epochs. Training loss gauges the model’s adaptation to the training data, while validation loss measures its adaptability to unfamiliar data. Epoch 4, highlighted in Table 3, was selected as the optimal model. This epoch shows a training loss of 0.0180 and a validation loss of 0.1896, which is the most favorable combination among all the epochs. Precision for this model stands at 0.9118, with a Recall of 0.9018, an F1-Score of 0.9098, and an impressive accuracy of 0.9952. Such metrics suggest a commendable rate of correct predictions by the model. It appears the model values stabilized between the 5th and 9th epochs.

**Table 3:** GNViT model training results.

Epoch	Train Loss	Train Acc	Val Loss	Val Acc
1	0.6599	82.38%	0.3354	88.34%
2	0.1042	96.06%	0.1882	93.15%
3	0.0289	99.16%	0.2120	93.03%
4	0.0180	99.52%	0.1896	95.31%
5	0.0190	99.46%	0.2611	93.15%
6	0.0530	98.38%	0.3492	90.38%
7	0.0522	98.56%	0.2754	92.79%
8	0.0404	98.77%	0.3604	92.79%
9	0.0831	97.50%	0.3167	91.23%
10	0.1173	96.36%	0.4084	90.14%

### 4.3. Performance analysis of the GNViT model on pest dataset without augmentation

**Fig 5:**
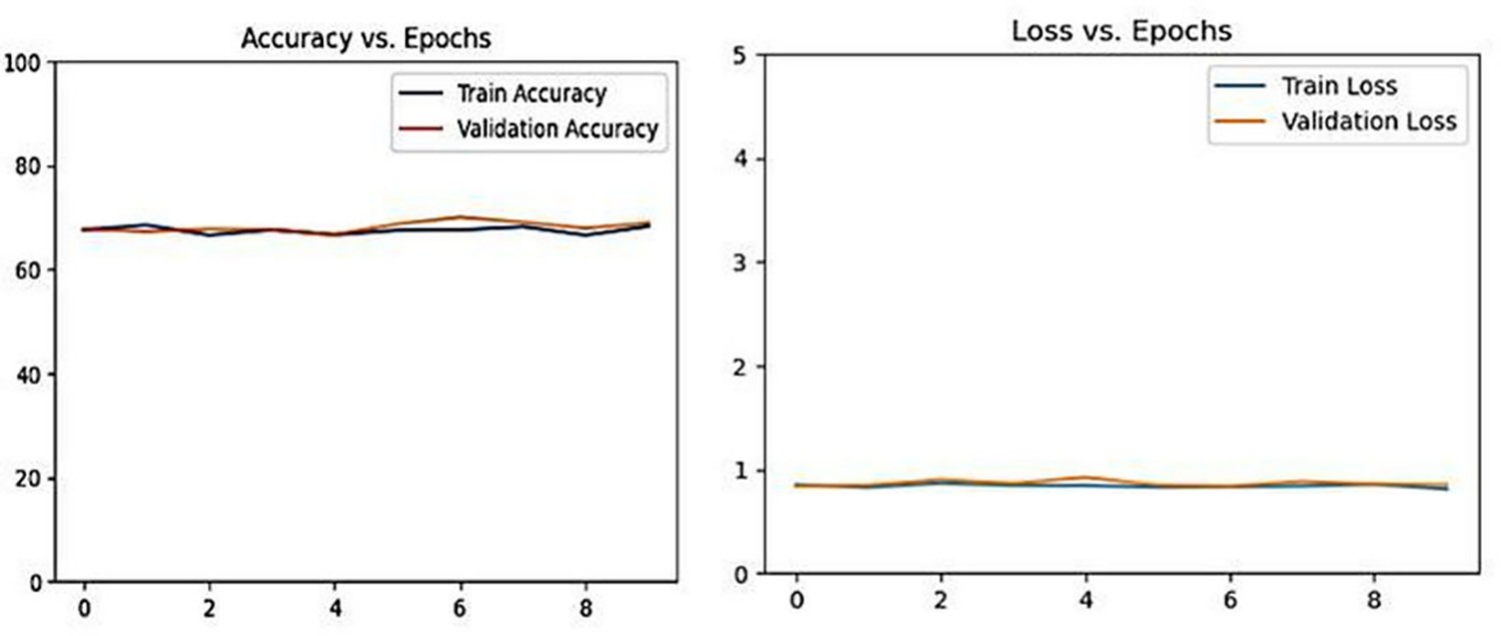
GNViT model performance on groundnut pest classification accuracy for 10 epochs without Augmentation.

During ten epochs, the GNViT model, devoid of Augmentation, showcases its performance using metrics from both the training and validation datasets, notably loss and accuracy. Fig 5 demonstrates GNViT model performance on Groundnut pest classification accuracy for 10 epochs without Augmentation. As training progresses, disparities in the training and validation scores surface, indicating potential convergence issues. The model performs best in the 7th epoch, with a validation accuracy of 70.07%. However, the inconsistent metrics throughout the epochs hint at potential enhancements or modifications that could be applied to the training approach.

### 4.4. Performance comparison of accuracy between GNViT and state-of-art methods

Table 4 presents the results of several models applied to the Pest dataset, including LeNet, AlexNet, GoogleNet, ResNet, EViTA + PCA + MFO, and the proposed GNViT, after training for a specific number of epochs.

**Table 4:** Comparison of accuracy between GNViT model and existing models.

Model	Accuracy (%)
LeNet [50]	66.00
AlexNet [50]	72.00
GoogleNet [50]	56.00
ResNet [50]	62.00
EViTA + PCA + MFO [50]	92.00
GNViT (Proposed Model)	99.52

The presented classification models achieved varying accuracies on the dataset—GNViT with an impressive 99.52%. EViTA + PCA + MFO performed well with an accuracy of 92.00%. However, the other models exhibited lower accuracies, i.e., ResNet reached 62.00%, GoogleNet scored 56.00%, AlexNet achieved 72.00%, and LeNet reached 66.06%. The accuracies of models such as LeNet, AlexNet, GoogleNet, ResNet, and EViTA + PCA + MFO are based on the findings reported in [50].

### 4.5. GNViT learning model performance on predicted images

The GNViT model performs well on correctly predicted images, indicating its accuracy in classifying groundnut pests. It randomly selects a subset of images from the test dataset and displays them in a grid format. For each image, the actual label is shown in green, and the predicted label is shown in red if it does not match the existing label, making misclassifications easily identifiable. This allows users to quickly inspect the model’s predictions and identify potential areas of improvement or errors. The visualization provides an intuitive way to understand how well the model performs. It can be used for debugging, fine-tuning the model, or gaining insights into its strengths and weaknesses. The GNViT model demonstrates impressive performance on correctly predicted images. The model exhibits high accuracy in classifying pests across multiple classes, with an accuracy of 99.52%. The model’s ability to accurately predict images indicates its proficiency in capturing and learning intricate patterns and features from pest images. The low loss value of 0.0165 further signifies the model’s capability to minimize prediction errors. These findings demonstrate how efficient the GNViT model is in correctly identifying and classifying groundnut pests, showcasing its potential for automated pest detection and classification.

The GNViT model boasts impressive performance metrics: a precision of 0.9193, indicating that 91.93% of its positive predictions are correct; a recall of 0.9189, suggesting that it correctly identifies 91.89% of all actual positive cases; and an F1-score of 0.9190, which is a harmonized metric reflecting the balance between precision and Recall. These closely matched scores hint at a model that maintains a good balance in its predictions, minimizing false positives and false negatives and showcasing robustness in its performance.

### 4.6. Discussion

The research used advanced computational methods, including the PyTorch framework and Vision Transformers (ViT), to tackle groundnut pest classification. Our models were optimized using the Adam optimizer for superior performance. Experiments were conducted on a GPU-enabled P-100 server with an i5 processor and 8GB RAM. We divided our data into 80% training, 10% validation, and 10% testing sets and trained our models over 10 epochs with a batch size of 64. After careful hyperparameter tuning, we chose a learning rate 0.0001 and a warm-up ratio of 0.1.

In our research, using data augmentation with the GNViT model led to a marked rise in training accuracy from 55.37% in the initial epoch to 98.46% by the tenth. Despite this success, an uptick in validation loss hinted at potential overfitting, underscoring the ever-present balance challenge in machine learning. Conversely, without data augmentation, the GNViT model exhibited inconsistencies between training and validation results, emphasizing the pivotal role of Augmentation, particularly when handling limited or imbalanced data sets.

In our research, the GNViT model distinguished itself as the leading architecture with an accuracy of 99.52%, surpassing other models. Its impressive precision of 91.18%, Recall of 90.18%, and F1-score of 90.98% underlined its robustness and balanced prediction capabilities. The model’s adeptness at discerning intricate patterns from pest images positions it as a potential cornerstone for automated pest detection in precision agriculture. Future work should address overfitting and the model’s practical applications in agricultural scenarios.

From Table 4 provides a comparative analysis of the performance of the GNViT model alongside several existing models for groundnut pest classification. Notably, while baseline models like LeNet and AlexNet achieve moderate accuracies of 66.00% and 72.00% respectively, more complex architectures like GoogleNet and ResNet exhibit lower accuracies of 56.00% and 62.00%. However, the EViTA + PCA + MFO model, integrating additional techniques, achieves a notable accuracy of 92.00%. Remarkably, the GNViT model outperforms all others with an impressive accuracy of 99.52%, showcasing the efficacy of the Vision Transformer architecture in handling the intricacies of groundnut pest images. These results underscore the significance of advanced model architectures tailored to the dataset characteristics, emphasizing the GNViT model’s potential for precise and reliable pest detection in agricultural settings.

The proposed study stands out as a beyond state-of-the-art initiative by pioneering the innovative application of the Vision Transformer (ViT) in agricultural pest classification, with a focus on groundnuts. The GNViT model, complemented by data augmentation techniques, achieved an impressive training accuracy of 99.52%, surpassing existing state-of-the-art methodologies, particularly in accuracy. This breakthrough represents a significant advancement in deploying deep learning models for efficient and reliable pest classification in agriculture. By leveraging cutting-edge technological solutions, the research makes a substantial contribution to mitigating crop losses and enhancing global food security amid challenges posed by population growth and limited agricultural land. The study’s distinctive feature lies in its adoption of the Vision Transformer model, a sophisticated and novel approach in deep learning, tailored to address the unique challenges of pest classification in groundnut crops.

The proposed GNViT model, employing deep learning models for pest detection in groundnut crops, faces several limitations. The most notable is related to overfitting and data augmentation. An increase in validation loss hinted at potential overfitting, a common challenge in machine learning where a model learns the training data too well, including its noise and anomalies, but fails to generalize effectively to new, unseen data. Additionally, the GNViT model showed inconsistencies between training and validation results without data augmentation, emphasizing the critical role of this process, especially when handling limited or imbalanced datasets. Addressing these issues, including overfitting and the necessity for effective data augmentation, is suggested as a focus for future work to enhance the model’s practical applications in agriculture.

However, the performance of GNViT model’s superior performance in groundnut pest classification, there are some key limitations. Primarily, the focus on accuracy as the sole performance metric excludes essential metrics like precision, recall, and F1-score, crucial for comprehensive evaluation, particularly in imbalanced datasets. Moreover, the discussion neglects addressing dataset biases, model generalization to unseen data, and lacks an in-depth analysis of computational efficiency and model complexity. The absence of considerations regarding the GNViT model’s interpretability further limits the evaluation’s depth. Addressing these limitations would enhance the overall assessment of the GNViT model’s efficacy in groundnut pest classification.

## 5. Conclusion

Our research has demonstrated the efficacy of the GNViT model in the realm of groundnut pest classification. With an unparalleled accuracy of 99.52%, it outperformed other architectures, solidifying its position as a reliable and robust tool for this task. Notably, its precision, Recall, and F1 score showcased its superior ability to make balanced predictions, minimizing false positives and harmful errors. Such adeptness in identifying intricate patterns from pest images accentuates its promise in revolutionizing pest detection methodologies.

While the GNViT model has exhibited outstanding performance, it is essential to address the observed overfitting, which could hinder its real-world applicability. Future research could delve deeper into integrating advanced regularization techniques or exploring architectures that inherently combat overfitting. Additionally, understanding the model’s behavior in diverse environmental conditions and with varied datasets will be crucial. Integrating the model into real-world agricultural setups, possibly through mobile applications or on-field devices, can pave the way for more targeted and efficient pest control strategies, catalyzing a new frontier in precision agriculture.
